# Supplementary material for: Collaborative intelligence in AI: Evaluating the performance of a council of AIs on the USMLE
Source: PLOS Digit Health. 2025 Oct 9;4(10):e0000787. doi: 10.1371/journal.pdig.0000787 (PMC12510544; doi:10.1371/journal.pdig.0000787)
Supplement: S2 File — (ZIP) [file pdig.0000787.s002.zip › S2 Data Raw data files containing unprocessed question inputs and LLM outputs including transcripts of deliberations/STEP3_Question-4-1_Type-MC-NJ.docx]

**Facilitator** **[Round 1]:** A 45-year-old woman comes to the office because she has noticed during the past several months that her fingernails have become white, increasingly brittle, and seem to lift off her nail beds easily. During this time, she also has had episodes of feeling warm and perspiring. Medical history is remarkable for intravenous drug use 25 years ago. The patient takes only a multivitamin. Last menstrual period was 3 weeks ago. She appears thin and mildly anxious. She is 170 cm (5 ft 7 in) tall and weighs 48.5 kg (107 lb); BMI is 17 kg/m² . Vital signs are temperature 37.0°C (98.6°F), pulse 106/min, respirations 20/min, and blood pressure 136/92 mm Hg. Physical examination of the hands shows a white discoloration of the nail beds. The remainder of the examination shows no abnormalities. Which of the following is the most likely diagnosis? (A) Chronic obstructive pulmonary disease (B) Diabetes mellitus (C) Endocarditis (D) Hyperthyroidism (E) Perimenopause

**AI_A:** RESTATING THE QUESTION: The question asks us to identify the most likely diagnosis for a 45-year-old female patient, who shows symptoms of onycholysis (nail lifting), episode of feeling warm, perspiring, low weight with lower BMI, high pulse rate, and high blood pressure. Her medical history includes intravenous drug use a quarter of a century ago. PLANNING: To get to a probable diagnosis, we should consider two assumptions. The first assumption would be associated with her past of intravenous drug use, possibly leading to endocarditis. The second assumption would focus on her symptoms that are suggestive of a possible hormonal or metabolic condition such as hyperthyroidism. CLINICAL REASONING: When we analyze the first assumption, the typical findings of endocarditis like heart murmurs and fever are absent. Also, isolated nail changes without other clinical features like Osler's nodes, Janeway lesions make endocarditis unlikely. Let's focus on the second assumption for now. The patient's constitutional symptoms, such as feeling warm, perspiring, increased pulse rate, and anxiety, align with hyperthyroidism. Hyperthyroidism, an excess production of thyroid hormones, can increase the body's metabolic rate leading to these symptoms. Additional signs include weight loss, which is corroborated by the low BMI indicative of being underweight. Onycholysis is also an associated sign of hyperthyroidism; it refers to nail lifting from the nail bed and tends to occur with nails' whitening and brittleness. CONCLUDING: Since the clinical picture doesn't support a diagnosis of endocarditis, diabetes, COPD, or perimenopause, the most suitable diagnosis would be hyperthyroidism. Diabetes mellitus or COPD does not explain her thin body habitus, high pulse, and high BP. Perimenopause might be a possibility due to her age, but it doesn't fully explain other observed signs and symptoms. RESPONSE: Therefore, the most likely diagnosis for this patient's condition is (D) Hyperthyroidism.

**AI_C:** RESTATING THE QUESTION: The patient, a 45-year-old female, presents with several symptoms: white, brittle fingernails lifting from nail beds, episodes of feeling warm and perspiring, a thin appearance, and mild anxiety. She doesn't have a preexisting condition apart from a history of intravenous drug use two decades ago, and her vital signs indicate a slightly increased pulse rate and blood pressure. The key question is which diagnosis fits this clinical picture best from the options given: chronic obstructive pulmonary disease, diabetes mellitus, endocarditis, hyperthyroidism, or perimenopause. PLANNING: The primary assumption here is that there is a connection between the above symptoms. An alternate assumption might be that some of these symptoms are causally disconnected. However, a more plausible assumption is to consider a disease state that could explain all symptoms. Among the choices given, hyperthyroidism and perimenopause could account for the various symptoms. CLINICAL REASONING: Hyperthyroidism often presents with symptoms like heat intolerance (feelings of warmth and perspiring), anxiety, an increased basal metabolic rate (leading to weight loss despite normal or increased appetite) which might be the reason why the patient is thin, and tachycardia (which could cause the pulse rate of 106/min). Hyperthyroidism can also occasionally cause hair and nail changes. Conversely, perimenopause could potentially explain the heat intolerance symptoms (as hot flashes are common in menopause), but it wouldn't usually account for the nail changes or the patient's thin appearance. Also, with her last menstrual period only three weeks ago, perimenopause seems less probable. CONCLUDING: The given symptom cluster, including white, brittle nails, feeling warm, increased pulse rate, weight loss, and anxiety, is more consistent with hyperthyroidism than with any of the other listed diagnoses. While the patient's history of intravenous drug use is a concern, endocarditis wouldn't explain the rest of her symptoms. RESPONSE: Therefore, the most likely diagnosis for this 45-year-old woman is hyperthyroidism (D). Her symptoms align closely with common presentations of this condition, making it the most probable explanation. Further diagnostic tests such as thyroid hormone level assessments would be useful to confirm this diagnosis.

**AI_D:** RESTATING THE QUESTION: The patient in question, a 45-year-old female, is presenting symptoms primarily concerned with changes to her fingernails: they have turned white, become brittle and easily separate from the nail beds. This is alongside episodes of warmth and sweating. The patient's medical history notes past intravenous drug use, which could potentially provide context for her condition. She's underweight based on her calculated BMI of 17 kg/m², and her vitals reveal elevated heart rate and blood pressure. With this information, we're asked to determine which diagnosis best fits her symptoms: chronic obstructive pulmonary disease, diabetes mellitus, endocarditis, hyperthyroidism, or perimenopause. PLANNING: Each diagnosis has some validity, given the accompnying symptoms. Chronic obstructive pulmonary disease (COPD) often associates with past drug use, but the lack of respiratory symptoms is unexpected. Diabetes mellitus might explain the nail symptoms and the sweating, but the lack of glucose-related issues and her low BMI are contrary to the usual presentation. Endocarditis could potentially link to the past IV drug use, but the absence of cardiac symptomatology rules it less likely. Hyperthyroidism corresponds to the presented symptoms including anxiety, high metabolic rate (thin body, heat intolerance, and sweating), change in nails, and increased heart rate. Perimenopause, a normal physiological process for her age, might cause hot flashes and perspiration, but it doesn't account adequately for the nail changes or the vital signs. CLINICAL REASONING: The thermal intolerance and sweating our patient is experiencing suggest a high metabolic state. Connected with her low BMI and anxiety, this could be indicative of hyperthyroidism, which results from an overactive thyroid gland that produces too much thyroid hormone, leading to a high metabolic state. Furthermore, this thyroid dysregulation can cause changes to the skin and nails, which would explain why the patient's nails are white and brittle—this is specific symptom called onycholysis is often seen in hyperthyroidism. CONCLUDING: Given the patient's mixture of symptoms that include those classically associated with hyperthyroidism (warmth, perspiration, nail changes, high heart rate and anxiety), it appears to be the most likely diagnosis out of the options provided. Other symptoms associated with the remaining diseases are lacking, making hyperthyroidism the most clinically sound and logically consistent answer. RESPONSE: The most likely diagnosis for this patient given her presentation and symptoms is (D) Hyperthyroidism.

**AI_B:** RESTATING THE QUESTION: To summarize, we have a 45-year-old woman who presented with complaints of changes in her fingernails such as whitening, brittleness, and easy lifting from the nail beds. Concurrently, the patient also reports feeling warm and experiencing episodes of perspiration. Her medical background reveals a history of intravenous drug use, dating back 25 years ago but currently she only takes multivitamins. She had her menstrual period 3 weeks ago and at the current moment appears to be thin and mildly anxious. Preliminary physical examination reveals a discoloration of her nail beds; the rest of the examination results found nothing abnormal. Our task is to find the most probable diagnosis of her condition from the given choices: Chronic obstructive pulmonary disease, Diabetes mellitus, Endocarditis, Hyperthyroidism, or Perimenopause. PLANNING: Let's evaluate each possible diagnosis based on the symptoms and findings we have. Chronic obstructive pulmonary disease (COPD) is generally associated with chronic smoking and chronic respiratory symptoms, none of which was reported by this patient. Diabetes mellitus generally presents with polyuria, polydipsia, unexplained weight loss and sometimes vision problems, and these symptoms do not match our patient's complaints. Endocarditis generally occurs in individuals who have a history of heart valve problems or after invasive procedures, and it typically presents with fever, malaise, new heart murmur, petechiae, or other vascular phenomena. Given the patient's history of intravenous drug use, it might be a possible diagnosis if she had the typically associated symptoms, which she does not. Hyperthyroidism often presents with signs and symptoms such as heat intolerance (matches with the patient's feeling of warmth), palpitations, weight loss despite normal or increased appetite, anxiety, increased sweating (possible match with patient's sweating episodes); but we would need more specific signs like goiter or exophthalmos to confirm. Finally, perimenopause could explain the patient's anxious feeling because women in perimenopause often experience mood swings and hormonal imbalance, but wouldn’t explain most of the physical symptoms. CLINICAL REASONING: Considering the likelihood of each possible diagnosis based on the patient's symptoms, hyperthyroidism appears to be the most plausible. This patient exhibits multiple symptoms that are commonly associated with hyperthyroidism, including heat intolerance, anxiety, and nail changes. In cases of hyperthyroidism, one may develop a disorder known as onycholysis, where the nails become loose from the nail bed, which fits the patient's description. Furthermore, the signs like unexplained weight loss (low BMI of 17 kg/m²), tachycardia (pulse 106/min), and high systolic blood pressure further supports the diagnosis of hyperthyroidism. CONCLUDING: While we cannot conclusively rule out other possibilities without further testing, the set of symptoms and clinical details described by the patient predominantly points towards the manifestation of hyperthyroidism. This condition would satisfactorily explain both the characteristic changes in the nails along with the episodes of feeling warm and perspiring, the apparent weight loss, increased heart rate and blood pressure, and her overall anxious demeanor. RESPONSE: Therefore, within the given context, the most likely diagnosis for this patient would be (D) Hyperthyroidism.

**AI_E:** (1) RESTATING THE QUESTION: The scenario presents a 45-year-old female who has experienced changes in her nails over the past months: they have turned white, have become brittle, and tend to lift from her nail beds. Concurrently, she feels warm and sweats excessively. Historical data tells us she was involved in intravenous drug use 25 years back and is currently consuming only multivitamins. Her last menstrual cycle was 3 weeks ago. Her physical appearance suggests thinness and mild anxiety. Her BMI is low at 17, indicative of being underweight. Vitals examination showed normal temperature, elevated pulse rate, and high normal blood pressure. On physical examination, her nails are discolored white, but other physical examination is normal. This patient's condition needs to be diagnosed based on the given options which are Chronic obstructive pulmonary disease (COPD), Diabetes Mellitus, Endocarditis, Hyperthyroidism and Perimenopause. (2) PLANNING: Given the clinical details, we can make a few assumptions. The first assumption, given the presentations of white and brittle nails, intermittent warmth, sweating, anxious appearance and low BMI, could coalesce towards a diagnosis of Hyperthyroidism. Another assumption could be Endocarditis given her history of IV drug use which presents a risk for such an infection. However, the lack of symptoms and signs relating to infection such as fever, heart murmur or septic emboli reduces the likelihood of this assumption. Diabetes mellitus could be considered with the diaphoresis, but other symptoms like polyuria, polydipsia and hyperglycemia in the clinical presentation are missing. The likelihood of COPD and Perimenopause can be reduced due to the lack of associated symptoms. The most clinically prioritized assumption to explore would be Hyperthyroidism due to the convergence of several associated symptoms in the clinical presentation. (3) CLINICAL REASONING: The constellation of symptoms in this patient suggests Hyperthyroidism. The feeling of heat intolerance and perspiration are signs of a hyperactive thyroid disorder, where there is an increased metabolic rate. Anxiety could be linked to this as well, as mood disturbances are pertinent in hyperthyroidism. The patient's low body mass index and thin appearance also support this suspicion, as weight loss is a common occurrence due to augmented metabolic activity. One significant physical feature in this case is the nails lifting off the nail beds, a condition known as onycholysis, which is seen in hyperthyroidism. Terry's nails, as characterized by white discoloration, while associated with liver diseases, can also occur in thyroid diseases. The reduced BMI, scant body weight, increased heart rate, and raised blood pressure are all physiological changes that can occur as a result of an overactive thyroid gland. The combined clinical evidence seems to indicate a hyperthyroid state. (4) CONCLUDING: Chronic obstructive pulmonary disease is unlikely without respiratory symptoms. Diabetes mellitus would usually present with symptoms of hyperglycemia which are absent here. Endocarditis would have other signs of infection or heart-related symptoms. Perimenopause could be plausible for her age group but would typically present with irregular menstrual cycle, which is not reported in this case. On balance and exception, hyperthyroidism appears to be the most correlating option here, aligning with most of her symptoms. (5) RESPONSE: Hence, considering her symptoms of increased metabolism such as heat intolerance, sweating, anxiety, low BMI, and onycholysis along with accompanying white discolored nails, the most likely diagnosis is Hyperthyroidism (D).

**Facilitator** The consensus among the AI responses is that the most likely diagnosis for the 45-year-old female patient is hyperthyroidism. This conclusion is based on her symptoms, which include onycholysis (nail lifting), episodes of feeling warm, perspiring, low weight with lower BMI, high pulse rate, and high blood pressure. These symptoms align with hyperthyroidism, a condition characterized by an excess production of thyroid hormones, which can increase the body's metabolic rate. The patient's history of intravenous drug use was considered, but the lack of typical findings of endocarditis, such as heart murmurs and fever, makes this diagnosis unlikely. Other potential diagnoses, such as chronic obstructive pulmonary disease, diabetes mellitus, and perimenopause, were also considered but were ruled out as they do not fully explain the patient's symptoms. Therefore, the consensus is that the most likely diagnosis for this patient's condition is hyperthyroidism. Further diagnostic tests, such as thyroid hormone level assessments, would be useful to confirm this diagnosis.
